# Role of Microbial Toxins in Neurodegenerative Diseases: Insights and Future Perspectives

**DOI:** 10.3390/biom16060790

**Published:** 2026-05-27

**Authors:** Alka Ashok Singh, Fazlurrahman Khan, Minseok Song

**Affiliations:** 1 Department of Life Sciences, Yeungnam University, Gyeongsan 38541, Republic of Korea; 2 Ocean and Fisheries Development International Cooperation Institute, Pukyong National University, Busan 48513, Republic of Korea; 3 International Graduate Program of Fisheries Science, Pukyong National University, Busan 48513, Republic of Korea

**Keywords:** neurodegenerative diseases, microbial toxins, oxidative stress, neurotoxicity, mycotoxins, bacterial toxins, fungal toxins, neurological disorders, therapeutic strategies

## Abstract

Neurodegenerative disorders, including Parkinson’s, Alzheimer’s, and multiple sclerosis, are significant global health issues characterized by escalating neuronal dysfunction and cognitive decline. Studies suggest that microbial toxins originating from fungi and bacteria may contribute to neurodegenerative processes by altering neuronal homeostasis in several ways. Toxins formerly associated with infectious diseases have now been associated with neuroinflammation, oxidative stress, and protein misfolding, all of which are common in neurodegenerative diseases. According to recent studies, microbial toxins generated by the gut microbiota may cross the blood–brain barrier and possibly contribute to neuroinflammatory cascades linked to the development of neurodegenerative diseases. The complex interplay of microbial metabolites, microbial responses, and mitochondrial dysfunction demonstrates the diverse character of neurodegenerative processes. This review delves into the current understanding of microbial toxins, which are produced by diverse bacteria and can have a direct or indirect impact on neuronal health via multiple signaling pathways. Understanding the signaling mechanisms of microbial and toxin-mediated neurodegenerative diseases could result in the development of effective alternative therapeutics for neurological disorders.

## 1. Introduction

Neurodegenerative disorders are a global public health concern characterized by the gradual decline of neuronal function and structure [1]. Parkinson’s disease (PD), Alzheimer’s disease (AD), amyotrophic lateral sclerosis (ALS), and Huntington’s disease (HD) not only impact individuals but also impose an additional burden on medical facilities and the public as a whole [2]. Despite substantial investigation, the exact causes and pathophysiology of several diseases are unknown [3], complicating the development of effective treatments and preventive strategies. Recent breakthroughs in neurobiology have shown a complex relationship between microbial toxins and neurological disorders, emphasizing the necessity for efficient preventive interventions [4]. Microbial toxins, traditionally considered infectious disease agents, are a type of toxin produced by bacteria [5], and fungi are recognized for their potential contributions to neurodegeneration [6]. These toxins have a variety of chemical structures and methods of action, which can alter neuronal homeostasis, cause inflammatory responses, and increase neurotoxicity [7]. Determining the involvement of microbial toxins in neurological disorders is a promising area of research, providing new perspectives into disease mechanisms and potential treatment targets.

Neurodegenerative disorders cause a gradual loss of neurons and synapses [8], leading to cognitive deterioration, motor dysfunction, and, subsequently, serious impairment [9]. AD, the most common type of dementia, is distinguished by the formation of beta-amyloid plaques as well as tau protein neurofibrillary tangles in the brain, which contribute to cellular death and cognitive decline [10]. PD is caused by the loss of dopaminergic neurons in the substantia nigra, which results in motor signs such as tremors and stiffness [11]. ALS damages motor neurons in the spinal cord and brain, causing muscle deterioration and immobility [12]. These conditions share characteristics such as misfolding of proteins, neural inflammation, oxidative stress, and mitochondrial dysfunction, all of which contribute to progressive neurotoxicity [13].

This review focuses on the five interrelated mechanisms—neuroinflammation, oxidative stress, protein misfolding and aggregation, mitochondrial dysfunction, and disruption of the blood–brain barrier—by which microbial toxins cause neurodegeneration.

Microbial toxins have long been explored for their roles in promoting infectious diseases [14,15]. For example, Clostridium tetani toxin and Clostridium botulinum toxin are well-known for their neurotoxic properties, which cause muscle paralysis by interrupting neural communication at neuromuscular junctions [16]. However, recent studies have indicated that microbial toxins may play essential roles in neurological diseases not associated with typical infection routes [17]. Multiple microbial toxins are being linked to neurodegenerative diseases via diverse mechanisms. For instance, by stimulating astrocytes and microglia in the central nervous system, lipopolysaccharide (LPS), which is part of the cell walls of Gram-negative bacterial cells, can cause neuroinflammation and oxidative stress in the central nervous system (CNS) [18]. This inflammatory reaction causes neuronal damage and increases disease progression, such as AD and PD [19]. Fungal toxins, such as those generated by *Candida albicans*, have been associated with protein misfolding and aggregation, a characteristic common to neurodegenerative disorders [20]. Toxins can trigger the aggregation of α-Syn and other proteins associated with PD, implying a link between fungal infections and disease progression [21].

Microbial toxins in the CNS stimulate innate immune responses, which recognize and combat neurodegeneration through pattern recognition receptors (PRR), such as toll-like receptors (TLR) [22]. The activation of these receptors causes the production of pro-inflammatory chemokines, cytokines, and reactive oxygen species (ROS), which enhance neuroinflammation and worsen neuronal injury [23]. Microbial toxins can potentially have a direct effect on neuronal function and survivability [24]. In particular, several bacterial toxins disrupt intracellular signaling pathways vital for the survival of neurons and synaptic activity. Toxins generated by molds and fungi harm neurons by altering cellular membranes and mitochondrial activity, resulting in energy depletion and apoptosis [25]. Furthermore, certain microbial toxins behave like prions, causing host proteins to misfold and aggregate.

Microbial toxins, particularly α-Syn, have been associated with neurological conditions and may contribute to disease progression by spreading protein abnormalities throughout the brain [26]. Recognizing microbial toxins as potential contributions to neurodegenerative disorders brings up novel therapeutic opportunities. Addressing microbial toxins and their subsequent impacts on neurological inflammation, protein aggregation, and neural survival is an intriguing approach to delaying disease progression and maintaining neuronal function [27]. Microbial toxins in neurodegenerative diseases can have neurotoxic effects on the central nervous system, potentially altering immune responses and protein clearance systems. Nanotechnology, gene therapy, and immunization could be used to target these toxins, potentially providing novel treatments [28]. The current review focuses on the function of toxins in neurodegenerative diseases, their modes of action, and the correlation between gut microbiome and toxin generation.

Even though there is mounting evidence linking microbial toxins to neurological disorders, there are still a number of significant unresolved issues. The majority of currently available research is observational and does not definitively determine whether toxin production and microbial dysbiosis are secondary effects of disease progression or causative factors. Additionally, it remains unclear exactly how microbial toxins affect neuroinflammation, protein aggregation, mitochondrial dysfunction, and the disruption of the blood–brain barrier. The diversity of gut microbiomes among individuals is another significant obstacle that complicates the identification of toxin profiles and microbial signatures unique to a given disease. The intricacy of host-microbiota interactions is further highlighted by contradictory results about the role of specific microbial species in neuroprotection versus neurotoxicity. Despite improvements in experimental models, there has been little conversion of these discoveries into therapeutic approaches that are applicable in clinical settings. Therefore, additional mechanistic research and long-term clinical studies are required to elucidate the role of microbial toxins in the development and course of neurological disorders.

## 2. Gut Microbiota and Toxin Production

The gut microbiota, a complex network of bacterial species, is shaped by neonatal changes as well as environmental events, which influence its makeup [29]. Under adverse circumstances, the gut microbiota can produce excessive toxins, resulting in inflammation and the breakdown of the intestinal barrier. Gut microbiota diversity is affected by environmental and genetic variables, with nutrition playing a significant role [30]. Based on the findings, dysbiosis and bacterial endotoxins in PD patients may produce gastrointestinal inflammation, enhanced permeability, and α-Syn misfolding [31]. When intestinal microbes break down amino acids, including choline, betaine, l-carnitine, tyrosine, phenylalanine, and tryptophan, they release additional uremic toxins. The type of diet that contains these amino acids determines how these toxins develop [30]. In general, the digestive tract’s microbial degradation of AA produces uremic toxins. For example, the distal colon produces p-cresol by breaking down tyrosine and phenylalanine. Meanwhile, gut bacteria like Escherichia coli convert tryptophan into indole and indole acetic acid [32]. Autism spectrum disorder (ASD) is a neurological ailment that impacts an individual’s perception of their surroundings [33]. The same toxin can be synthesized by gut bacteria without being metabolized, and vice versa. Several distinct studies have demonstrated the bacterial origin of compounds, including phenol, phenylacetic acid, indole-3-acetic acid, p-cresyl sulfate, trimethylamine, trimethylamine-N-oxide, hippuric acid, and phenol [34,35,36,37]. Although ASD is primarily regarded as a neurodevelopmental disorder rather than a neurodegenerative disease, it is discussed here because there is mounting evidence connecting altered neurodevelopment and dysfunction of the gut–brain axis to gut microbiota dysbiosis, microbial toxin production, and neuroinflammatory pathways. Researchers have found that some bacteria, such as *Oxalobacter formigenes*, *Campylobacter upsaliensis*, *Desulfovibrio* sp., *Brevundimonas* sp., *Helicobacter pylori*, *Campylobacter coli*, *Desulfovibrio piger*, and *Phascolarctobacterium faecium*, can produce over 14 different toxins; nonetheless, they cannot all metabolize the same toxins. On the other hand, the following microorganisms are incapable of synthesizing toxins and can only metabolize over seven of them. *Clostridium sporogenes*, *Klebsiella oxytoca, Escherichia coli*, *Lactobacillus ruminis, Pediococcus acidilactici*, *Parvimonas micra*, *Listeria grayi*, *Listeria innocua*, and *Enterococcus faecalis*, Ruminococcaceae bacterium, are a few examples of bacteria [38].

Furthermore, it was discovered that uremic patients’ intestines had noticeably higher overall concentrations of *E. coli* and *Klebsiella* [39]. It should be emphasized that bacteria have the ability to eliminate dangerous solutes by using uremic retention solutes as nutrition. Certain bacteria exhibit the ability to express specific enzymes, such as urease (*Pseudomonas* spp.), which catalyzes the breakdown of urea, or urate oxidase (*Clostridia* spp.), which oxidizes uric acid [40]. When released into the gut, toxic chemicals like creatinine and oxalate are consequently digested by bacteria [41,42]. Within the normal microbiota, species belonging to the genera *Bifidobacterium*, *Enterococcus*, *Oxalobacter*, *Eubacterium*, and *Lactobacillus* have the ability to degrade oxalate, hence reducing its accumulation in the uremic zone [43].

**Figure 1:**
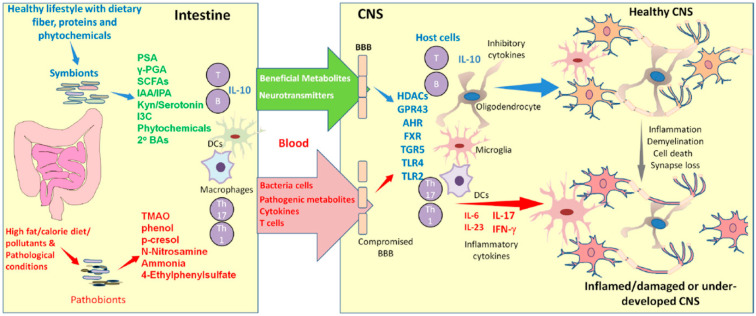
The modulation of CNS development and inflammatory responses by metabolites derived from the pathogenic and non-pathogenic microbial species in the gut. Reprinted from [44], Copyright @2021 by the author and Springer Nature.

The complex relationship between the bloodstream, the CNS, and the gut (intestine) is depicted in Figure 1, which shows how brain and gut health are influenced by one another. It primarily examines the gut microbiota (the community of bacteria in the intestine) and how it affects inflammation and neuronal function in the CNS. In contrast, a poor diet and environmental contaminants stimulate the growth of pathogenic bacteria, which create toxic compounds that can penetrate the blood–brain barrier (BBB), causing inflammatory and degenerative impacts in the central nervous system. Interleukin-10 (IL-10) is a cytokine that has anti-inflammatory characteristics and aids in immune response suppression, which helps shield the brain. Pro-inflammatory cytokines such as interleukin-6 (IL-6), IL-17, and interferon-gamma (IFN-γ), however, become active when the central nervous system is weakened. These cytokines worsen demyelination (damage to the covering that protects neurons), increase inflammation, and contribute to cell death and damage to neurons.

## 3. Microbial Toxins Implicated in Neurodegenerative Diseases and Their Action Mechanisms

The connection between microbiota and diseases like cancer, diabetes, and neurological disorders has been the subject of much research in recent decades. Gastrointestinal abnormalities, including diarrhea, constipation, abdominal pain, and barrier disruption, are also present in patients with ASD, and their severity is correlated with such abnormalities [45]. There is a correlation between greater intestinal permeability and higher behavioral severity in very young children who show signs of autism spectrum disorder [46]. There is a correlation between elevated blood levels of toxins and bacterial products, which are generated by increased intestinal permeability, and immune responses, which may result in reduced cognitive function and social behavior [47,48]. ASD-associated microbiota in the human gut have the potential to exacerbate ASD behavior in mice. There was a hypothesis that certain bacterial taxa and metabolites might have an effect on the autism spectrum disorder behavior of mice that had a human microbiome [49]. According to recent research, dysregulated immune responses and altered gut microbiota composition are frequently linked to ASD [47,50,51,52,53]. It should be acknowledged that the majority of evidence linking microbial metabolites and gut microbiota dysbiosis to neurodevelopmental and neurodegenerative outcomes is derived from animal models [54]. Although individuals with ASD and other neurological disorders have been found to have altered microbial composition and metabolite profiles, direct causal relationships in human populations are still not well established. To determine whether these microbial changes are secondary disease-associated changes or directly contribute to disease pathogenesis, more longitudinal and interventional research is needed. Children with GI problems and ASD were shown to have inadequate amounts of the taxa unclassified Veillonellaceae, *Coprococcus*, and *Prevotella* [55]. In addition, a meta-analysis discovered that children with autism spectrum disorder had lowered counts of *E. coli*, *Bifidobacterium*, *Enterococcus*, and Bacteroides but increased levels of *Ruminococcus*, *Lactobacillus*, and *Faecalibacterium* [51]. The feces of children diagnosed with autism spectrum disorder (ASD) were found to include higher levels of the *Clostridium histolyticum* group (*Clostridium* clusters II and I), which are known to possess the ability to create poisons [52]. Since it has been proven that the reduction of these Clostridia levels via the use of vancomycin may alleviate the symptoms of autism spectrum disorder (ASD), this suggests that bacteria belonging to the *C. histolyticum* group may contribute to ASD-associated symptoms [56].

**Figure 2:**
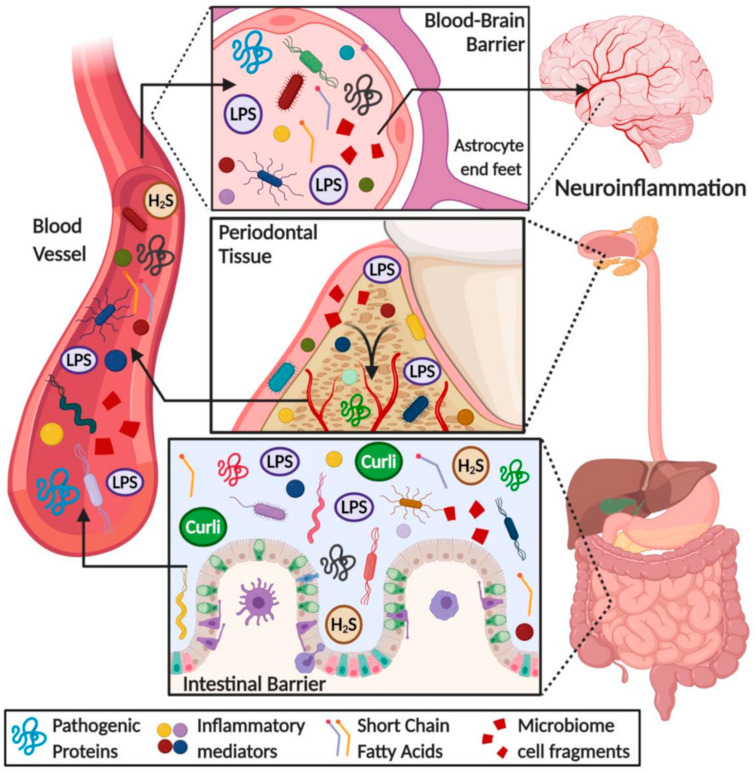
Function of the oral and intestinal microbiota in neurodegenerative disease neuroinflammation. Increased gut-blood barrier permeability allows microbial products like LPS, short-chain fatty acids, H_2_S, bacterial fragments, and amyloid-like proteins (like curli) to enter the systemic circulation and cross the blood-brain barrier into the central nervous system. Solid arrows indicate the translocation pathways of these metabolites across compromised barriers; dashed lines highlight the magnified anatomical regions. Color-coded icons represent pathogenic proteins, inflammatory mediators, and short-chain fatty acids as defined in the key. Reprinted from [68], Copyright © 2020 by the authors. Licensee MDPI, Basel, Switzerland.

Certain bacteria create proteinaceous toxins that damage the host’s neurological system Table 1. The general structure of these toxins is often similar, consisting of multiple subunits that activate intracellular or cell-surface receptors. Opportunistic pathogens can produce these toxins and live in the commensal community for extended periods without causing obvious disease in the brain or gut [7]. Numerous *Clostridium* species are recognized to generate a wide range of toxins, including enterotoxins, epsilon toxin, lethal toxin, and toxin B. These toxins can cross the BBB (Figure 2), disrupt systemic circulation, inhibit the release of neurotransmitters, and/or reduce the viability of neurons over a spectrum of a variety of target organs, including the gut and the hippocampus [7,24,57,58,59]. The toxins produced by *Staphylococcus* and *Bacillus* species, cereulide and staphylococcal enterotoxins, stimulate the vagus nerve, delivering messages to the brain that cause vomiting and other symptoms of illness [60,61,62]. A class of proteins known as amyloids is produced by other species, including *Salmonella* and *Escherichia* spp. These proteins aggregate in the intestine and have the potential to migrate to the brain in a manner resembling prion disease. They may also play a role in neurodegeneration, as seen in conditions like PD and AD [63,64,65,66]. Based on the fundamental knowledge of the gut–brain axis, research is still being done to determine which brain cells are impacted directly or indirectly by particular bacterial metabolites. It will take a lot of effort to methodically prove that these chemical messengers made from gut bacteria affect the growth or operation of particular brain cells. Here, we provide a summary of the available data suggesting that gut microbiota metabolites could have an impact on brain cells [67].

**Table 1:** Role of different types of toxins produced from diverse microorganisms in neurodegenerative diseases.

Microbial Toxins	Sources	Mechanism in Neurodegeneration	Associated Neurodegenerative Diseases	Level of Evidence	References
Lipopolysaccharide (LPS)	Gram-negative bacteria	Induces neuroinflammation and oxidative stress via microglial activation	Alzheimer’s disease, Parkinson’s disease	Moderate (preclinical + associative human evidence)	[69]
Beta-amyloid	Produced by bacteria and fungi	Aggregates into plaques, disrupts synaptic function	Alzheimer’s disease	Moderate evidence	[70]
Mycotoxins (e.g., aflatoxins)	*Aspergillus*, *Penicillium*	Disrupts cellular membranes’ mitochondrial function, inducing apoptosis	Neurotoxicity leading to cognitive impairment	Moderate evidence	[71,72]
Prions	Various sources, including fungi	Induce misfolding of host proteins, propagate protein aggregation	Prion diseases (e.g., Creutzfeldt-Jakob disease)	High evidence	[73]
Botulinum toxin	*Clostridium botulinum*	Inhibits neurotransmitter release, leading to muscle paralysis	Botulism	High evidence	[74]
Tetanus toxin	*Clostridium tetani*	Blocks inhibitory neurotransmission, causing muscle rigidity and spasms	hyperactivity and dysfunction in motor neurons,	High evidence	[75]
Staphylococcal enterotoxins	*Staphylococcus aureus*	Activates immune response, exacerbates neuroinflammation	Multiple sclerosis, Alzheimer’s disease	Moderate evidence	[76,77]
Shiga toxins	*Shigella dysenteriae*	Induces apoptosis in neurons, disrupts protein synthesis	Hemolytic-uremic syndrome, neurological sequelae	High evidence	[78]
α-Synuclein-like proteins	*E. coli*	Antibacterial proteins imitate or engage with human αSyn, encouraging its accumulation and dissemination.	Parkinson’s Disease	High evidence	[79]

### 3.1. Neuroinflammation: Contribution of Microbial Toxins to Neuroinflammatory Processes

**Figure 3:**
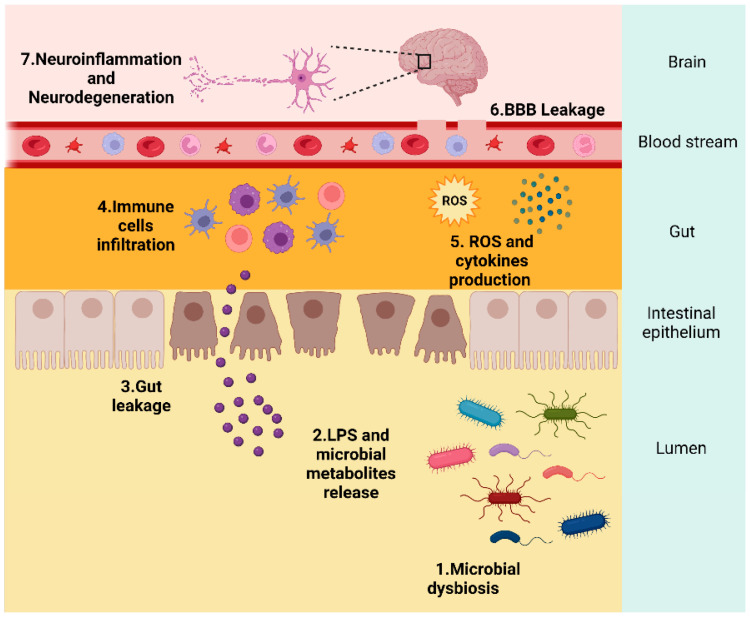
The role of gut microbiota and metabolites in neuroinflammation and neurodegeneration. (1) Gut microbiota dysbiosis causes an increase in the production of (2) LPS and several other metabolites, which (3) damage intestinal epithelial cells and gut permeability, allowing (4) immune cell infiltration, which produces (5) ROS and proinflammatory cytokines that enter the bloodstream and affect the brain. (5) Due to inflammation and the formation of a significant level of ROS, the BBB is impaired, eventually leading to (6) neuroinflammation and neurodegeneration. Reprinted from [117], Copyright© 2022 by the authors. Licensee MDPI, Basel, Switzerland.

According to epidemiological data, Figure 3 shows that chronic microbial infections are linked to neurological issues. The molecular complexity of cell-organ connections during microbial infections makes the underlying processes unclear [80]. Direct translocation of pulmonary bacteria and their soluble constituents may breach the lung alveolar-capillary barrier [81], enter the circulation, and utilize the BBB to reach the brain. Few studies have been done on direct translocation despite it being a possible lung-brain axis mechanism. Pseudomonas aeruginosa, a pneumonia-causing bacterium, may enter the bloodstream by damaging alveoli and introducing toxins to lung epithelial cells [82,83].

Interestingly, *P. aeruginosa* may cause meningitis and cerebral edema [84]. Another study indicates how pulmonary microbiomes influence CNS autoimmunity and MS [85]. The research found that lung bacteria’s cell wall components continually stimulate brain immune cells, polarizing brain-resident microglial cells toward a type I interferon (IFN) signature. Strong evidence is that the vagus nerve-mediated route and systemic circulation allow gut-central nervous system connection [86,87]. Even though these relations are largely beneficial, research into the connection between the human gut and brain has lately gained pace, demonstrating how vital the gut microbiota is in influencing behavior, stress reactions, and even certain mental diseases [47,88]. Bacterial compounds activate innate immune responses, causing an elevation in inflammatory cytokine levels [89]. Numerous studies have demonstrated that bacteria and the chemicals they generate can disrupt the BBB associated with various disorders [90,91,92].

BBB promotes TNF and permeability in animal sepsis models. Elevated TNF levels are associated with increased BBB permeability in animal sepsis models, suggesting that systemic inflammation is the primary driver of both TNF release and barrier disruption. [93]. Meningitis germs cross the BBB via binding to the brain endothelium with cell wall components or bacterial pili [94]. Some CNS-tropic bacteria may easily traverse the BBB, whereas others cannot [95]. Brain endothelial cells expressing the toll-like receptor (TLR) may transport LPS and LTA from Gram-negative and gram-positive bacteria to the central nervous system [96]. Combining LPS with other neuropathological stimulants like Aβ or cytokines might cause neuronal-glial cell inflammation and brain dysfunction in NDs [97,98].

The *P. gingivalis* main toxins, cysteine protease or gingipains, impair adaptive immunity and escape detection. Bacteria may also move from the oral cavity to the CNS and use gingipain activity to cause AD owing to immune suppression and tissue death. Numerous studies show that *P. gingivalis* and Aβs co-localize in the brain and in vivo [99,100,101]. Recent research suggests oral *P. gingivalis* injections may lead to p-tau protein, Aβ aggregation, and microbial brain invasion in mice [102]. In mouse research, blocking gingipain decreased tau tangles, Aβ plaques, neuroinflammatory responses, and neuron death. AD and bacterial infection research have advanced by discovering small-molecule gingipaine antagonists. Serum antibodies to periodontal bacteria such as *Eubacterium nodatum*, *Actinomyces naeslundii*, and *Prevotella intermedia* are greater in AD samples before AD occurs [103,104]. Spirochetes, another major periodontitis cause, may penetrate the CNS and are linked to AD [105]. Genetic and immunological components from oral Treponema species, *socranskii*, and *pectinovorum* were found in Alzheimer’s disease brain tissue [106].

About 10% of the 70,000 known fungal species affect the brain, with 300 of those species potentially detrimental to human health [107]. Proteomics and genomics investigations have revealed that the brain tissues of Alzheimer’s patients contain DNA and fungal proteins [108]. Bacterial species found in the CNS coexist with fungus taxa *Botrytis*, *Candida*, *Fusarium*, and *Malassezia* in the disease’s samples, indicating a possible danger to PD pathophysiology from combined bacterial and fungal infections [109]. Furthermore, the fungus species *Malassezia* has been discovered in MS patients [110], and it may enter the central nervous system through macrophages [111]. The Trojan horse process, which involves transcellular and paracellular migration, allows fungi to infiltrate the central nervous system via the BBB, promoting endothelial cell transcytosis and transport-mediated phagocytosis associated with Trojan horses [112,113,114]. Fungal proteins interact with proteins in the BBB through both transcellular and paracellular routes, facilitating their translocation across brain microvascular endothelial cells, as demonstrated by *Cryptococcus* neoformans [115]. *Candida albicans* incorporation begins when a heat shock protein in the brain endothelial interacts with an agglutinin-like protein precursor (ALs3) on the cell surface adhesion [116].

### 3.2. Oxidative Stress: Impact of Toxins on Oxidative Stress Pathways in Neurodegeneration

It has been established that oxidative stress is a common factor in neurodegenerative diseases. The mitochondria are an endogenous generator of ROS, which is a major cause of oxidative stress [118]. The term “oxidative stress” refers to the disturbance of the redox signaling pathway in cells as a result of a higher concentration of ROS than antioxidants [119]. Surprisingly, the CNS has a high oxygen need but a relatively low concentration of the enzymes needed to convert a variety of oxygen-based reactants into harmless species [120]. Conversely, the CNS is abundant in polyunsaturated fatty acids, which are easily oxidized by toxic oxygen compounds [121]. The BBB, which is intended to shield the brain from toxins by preventing their diffusion into neurons and glia, is another drawback. It also hinders or lessens the brain’s ability to absorb some antioxidants, such as vitamin E. 6-hydroxy-dopamine (6-OHDA), a recognized neurotoxin, is formed when dopamine is oxidized [122]. When consumed in extremely high doses, the stimulants methamphetamine (METH) and 3, 4-methylenedioxymethamphetamine (MDMA) also produce dopaminergic neurotoxicity [123]. Research suggests that the synthesis of deleterious oxygen-based radicals, especially from dopamine (DA), may represent a major contributor to methamphetamine’s neurotoxicity [124]. This notion is confirmed by research demonstrating that transgenic mice with increased superoxide dismutase activity are resistant to METH-induced toxicity [125]. Moreover, METH has been shown to cause a time-dependent rise in hydroxyl radicals in the brain [126], produce oxidative stress [127], and produce free radicals [128]. According to previous investigations, METH could damage the DAergic system by two key pathways: first, by causing hyperthermia, and second, by producing oxygen-mediated radicals and oxidative stress. These routes may interact with one another or influence each other, resulting in damage [123].

**Figure 4:**
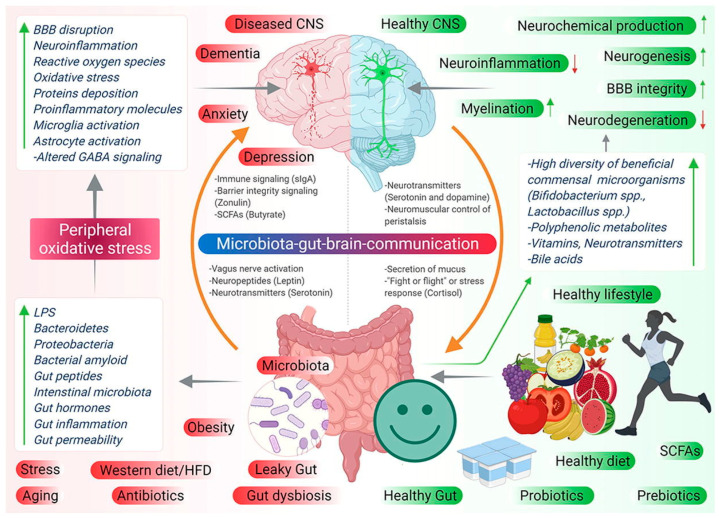
Understanding the link between oxidative stress and the gut microbiota in terms of neurodegeneration and neuroprotection. The colors and directional arrows represent pathological (red) versus healthy (green) pathways and their respective directional regulation as labeled. Reprinted from [119], Copyright @ 2022 by author and Elsevier B.V. on behalf of Cairo University.

The Microbiota–Gut–Brain Axis demonstrates the relationship between gut health and mental and cognitive functioning in Figure 4, which highlights this relationship. A balanced diet, probiotics, and prebiotics support a healthy gut environment that fosters the growth of good microorganisms such as *Lactobacillus* and *Bifidobacterium*, which increase neurogenesis and neurochemical production (dopamine, serotonin, etc.), decrease neuroinflammation, and preserve the integrity of the BBB. Avoiding neurodegenerative diseases and maintaining cognitive abilities leads to a healthy CNS.

### 3.3. Toxin-Induced Protein Misfolding and Aggregation in Neurodegeneration

Due to protein homeostasis changes, many neurodegenerative diseases may be caused by protein misfolding and the aggregation of many proteins into insoluble fibrils [129]. All these disorders have high-ordered insoluble fibrils formed by mutant proteins in intra- or extracellular aggregates. The protein’s identity determines which neurons are pathologically affected and each disease’s symptoms. Protein aggregation reasons are still being investigated [130]. Numerous annular species in proteofibrobrillar AβARC, resulting from the ‘Arctic’ mutation in amyloid precursor protein, imply a membrane permeabilization-mediated mode of plaque-independent neurotoxicity. These species mimic bacterial cytolytic pore-forming toxins. Protofibrillar Aβ species with the Arctic (AβARC) mutation in the amyloid precursor protein, a variation that is known to accelerate the formation of protofibrils. The rapid and increased formation of protofibrillar intermediates in PD-linked mutant αSyn may indicate that these are neurotoxic species, similar to bacterial pore-forming toxins, suggesting a potential toxic-mediated neuronal death mechanism involving membrane permeabilization [131]. Soluble αSyn oligomers increase in PD brains, and polyunsaturated fatty acids enhance their production [132]. Next, dissect misfolded protein-related pathogenic pathways. Membrane permeabilization via a channel mechanism known for microbial toxins may be part of AD, PD, and HD pathogenic pathways [131,133].

A cytotoxic state known as oxidative stress arises when there is a reduction in the antioxidant capacity of the cell and an increase in the intracellular overproduction, or buildup, of reactive oxygen species. Hydrogen peroxide (H_2_O_2_) and Superoxide (O_2_) are two of the most prevalent types of ROS. Furthermore, the formation of reactive nitric oxide (NO) species is linked to H_2_O_2_. These species can combine with O_2_ to produce peroxynitrite (PN), an incredibly potent oxidizing agent [134]. CNS microglia generate ROS through intracellular peroxidases, mitochondrial oxidative activities, and Nicotinamide Adenine Dinucleotide Phosphate Hydrogen (NADPH) oxidase activity on the cell membrane [135,136].

AD has been reported to be linked to a number of infection sources, including bacteria, viruses, and fungi [137]. The development of microbiome dysbiosis or infection with many harmful bacterial species may have a role in the pathophysiology of AD by inducing robust inflammatory reactions or assisting in the synthesis of Aβ [138,139]. Unexpectedly, bacterial infections can also result in persistent systemic inflammatory responses that damage neurons and accumulate Aβ/tau, which aids in the onset and development of AD. There are five to ten times as many bacteria in the brains of AD patients as in the brains of healthy people; the distribution and makeup of the bacteria vary as well [140]. Furthermore, it has been documented that Gram-negative bacteria are able to penetrate the BBB and support the development of tau hyperphosphorylation, Aβ buildup, and neuroinflammation in the brain [141]. Gram-negative bacteria, such as *Chlamydophila pneumonia* and Proteobacteria, have been linked to severe systemic inflammation and the development of AD [142,143]. Byproducts from Gram-negative bacteria, including LPS, capsular proteins, fibrillin, and flagellins, can reach the brain and cause tau and Aβ pathology, as well as neuroinflammation [144].

LPS is a potent endotoxin found primarily in the outer membranes of Gram-negative bacteria [145]. The immune system responds sensitively to excessive quantities of LPS, which can cause sepsis and septic shock [146]. Furthermore, sepsis caused by LPS increases the likelihood of developing AD and cognitive impairment [147,148]. Notably, the level of LPS in the plasma of AD patients is 3 to 6 times greater than that of normal; in AD animal models, the level of LPS in the blood is roughly 3 times higher than normal as well [145,149,150]. The pro-inflammatory immunomodulation brought on by LPS is thought to have a lethal impact on AD pathogenesis [151].

**Figure 5:**
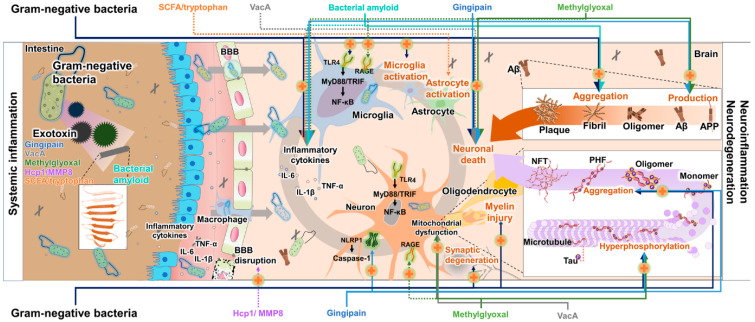
Gram-negative bacteria cause neuronal death by triggering the neuron’s TLR4 signaling pathway. They also create a variety of exotoxins that can penetrate the BBB and affect the pathophysiology of AD, such as gingipain, MG, bacterial amyloid, and VacA. Aβ synthesis is enhanced by gingipain and MG, whereas Aβ aggregation is encouraged by bacterial amyloid. Neurofibrillary tangles are caused by tau hyperphosphorylation, likewise fueled by these bacteria and poisons. Furthermore, they cause neurodegeneration and neuronal death by inducing neuroinflammation by activating microglia and astrocytes, which release inflammatory cytokines like IL-6, IL-1β, and IL-18. Reprinted from [69], Copyright @ 2021 by author(s) and Springer Nature.

The hydrophobic character of the aromatic amino acid peptides that make up the amyloid main sequence produced from amyloid precursor protein (APP) causes amyloid monomers to self-aggregate over time, forming dimers, oligomers, and fibrils. Neurodegenerative diseases like AD, PD, and prion disease are impacted by significant inflammatory reactions and neurodegeneration resulting from amyloid deposition in the brain [152]. Remarkably, a number of investigations have demonstrated the existence of amyloids made by bacteria [152,153,154] (Figure 5). The ability to form aggregates is one of the many physicochemical characteristics that bacterial amyloids share with the amyloids derived from humans [64,155]. Curli is a widely known Gram-negative bacterial amyloid [156]. It is a crucial component of the extracellular matrix biofilm that some enterobacterial strains, such as *E. coli*, create [157]. Remarkably, the bacterial amyloid and Aβ can attach to the same receptor because of their structural similarities. For instance, the bacterial amyloid from curli can attach to the TLR2-TLR1-CD14 (Toll-like receptor 2-Toll-like receptor 1-cluster of differentiation 14) complex, just like Aβ can. This binding helps to activate the NF-κB pathway, which is mediated by the activated B cells’ kappa light chain enhancer [158]. These results indicate that Gram-negative bacteria’s compounds may cause Aβ to be produced and aggregate, which could impact the development and course of AD and PD.

Additionally, pattern recognition receptors such as TLR-2 and 4 are impacted by exotoxins. Thus, bacterial imbalance and gut exotoxins impact neuronal cells’ function, which causes neuroinflammatory reactions, including microglial cell activation [159]. Inflammatory cytokines are released more frequently in response to gingipain and Gram-negative bacteria, which can cause neuronal death by stimulating the neural TLR4 signaling pathway, emphasizing the role of these bacteria in neurodegeneration [69].

### 3.4. Protein Aggregation

α-Syn (α-Syn), a 14 kDa protein linked to Parkinson’s disease, is common in human neural tissues. α-Syn maintains synaptic vesicles in presynaptic terminals; however, its mechanism is not fully known [160]. Although it is frequently seen in neurological diseases, protein aggregation is also linked to phenotypic plasticity in a wide range of species, including yeasts [161]. Phosphorylation at S129 of α-Syn is known to create aggregates characteristic of synucleinopathies; the role of phosphorylation in the biology and pathology of the protein remains debatable [162]. Phosphorylation was reported to decrease α-Syn toxicity and inclusion development in studies investigating the potential of budding yeast. Lewy bodies (LBs), a proteinaceous inclusion that is the primary cause of PD and other synucleinopathies, are characteristic pathological features of αSyn [163]. Interestingly, cells expressing S129A α-Syn had a lower clearance of α-Syn inclusions, which is correlated with inadequate autophagy activation. The discovery that phosphorylation affects cells’ capacity to remove α-Syn inclusions offers new light on the potential role phosphorylation may play in synucleinopathies. It also raises the possibility that posttranslational modifications serve as switches that cells use to regulate the aggregation and removal of important proteins, creating new opportunities for the development of treatment approaches for these debilitating conditions [164]. Iron levels in PD brains are pretty high and rise as the disease progresses [165]. Elevated iron levels may result from sequestration by eosinophilic protein aggregates, and iron has also been linked to αSyn aggregation promotion [166].

Fungal metabolites, known as mycotoxins, can infect humans and animals and cause a number of diseases. An additional connection between these fungus-derived metabolites and neurodegenerative diseases is becoming more and more apparent Table 2 [72]. Since fungi are found throughout the world, there is concern about people being exposed to their toxic secondary metabolites [167]. Mycotoxins are also secondary metabolites that some fungus species produce [168]. However, a growing body of research indicates that at least some mycotoxins are linked to neurodegenerative conditions like multiple sclerosis (MS), amyotrophic lateral sclerosis (ALS), Parkinson’s disease (PD), and Alzheimer’s disease (AD). The significance of evidence linking mycotoxins to specific neurodegenerative diseases varies widely. While mechanistic studies in cell culture systems and animal models have revealed biologically plausible pathways involving oxidative stress, mitochondrial dysfunction, neuroinflammation, and protein aggregation, direct epidemiological and clinical evidence in humans for most mycotoxin-disease associations remains limited. As a result, rather than being definitively causal, these associations should now be understood as hypothesis-generating.

Multiple sclerosis (MS): MS, a neurological disorder caused by the demyelination of neurons in the CNS, has been linked to mycotoxins [177]. MS appears to be caused by both genetic predisposition and environmental factors, with mycotoxins perhaps having an important role as part of the environmental “eco-exposome.” Mycotoxins like fumonisin B1 (FB1) may contribute to MS by altering sphingolipid production in neurons, resulting in demyelination [178,179]. Furthermore, a gliotoxic component detected in MS patients’ cerebrospinal fluid (CSF), probably connected to mycotoxin exposure, may promote glial cell death, compromising the BBB and activating an immunological response that further destroys myelin. While mycotoxins may not be the leading cause of MS, they are likely to work in tandem with hereditary and age-related variables to accelerate disease development [180,181,182].

Amyotrophic lateral sclerosis (ALS): There is suggestive evidence that plant-associated mycotoxin producers have a part in the development of amyotrophic lateral sclerosis (ALS), as shown by research that indicated an increased risk of ALS for farm workers and athletes who played on grass [183]. A number of fungi belonging to the genera *Fusarium*, *Trichoderma*, *Botrytis*, *Candida*, *Cryptococcus*, *Malassezia*, and *Penicillium* were found in the brain and cerebrospinal fluid (CSF) of individuals with amyotrophic lateral sclerosis (ALS) by the use of slot-blot, polymerase chain reaction (PCR), and proteomic analysis [184]. However, these fungi were not found in samples produced by healthy controls. In the majority of cases of amyotrophic lateral sclerosis (ALS), individuals had mixed fungal infections with many types. This evidence may suggest that a variety of fungi are capable of causing pathomechanisms that lead to amyotrophic lateral sclerosis [185]. ALS is characterized by an excessive release of glutamate (Glu) from neurons, which leads to excitotoxicity via the overactivation of Glu receptors and eventually ends in the death of motor neurons [186]. This is a well-known hallmark of ALS. Additionally, Glu is responsible for translocating the 43 kDa transactive response DNA binding protein (TDP-43) into the nucleus. This process results in the formation of cellular inclusion bodies, a prominent clinical feature of sporadic amyotrophic lateral sclerosis (ALS). Because of the relationship between TDP-43 and ALS [187], this is in line with the multi-ubiquitinylation of TDP-43. It is also possible that the mutant Copper/Zinc Superoxide Dismutase 1 (Cu/Zn-SOD1) in familial ALS patients may work in conjunction with the toxins [188]. This is because an increase in Glu causes the mutant protein to become more toxic. A minor increase in glutamate levels brought on by mycotoxins, which normally would not be sufficient to cause amyotrophic lateral sclerosis (ALS), may thus already be accountable for neurotoxic effects and muscle atrophy in individuals who have a hereditary vulnerability to the disease [189].

Parkinson’s disease (PD): In the 1980s, aflatoxins had been used in bioweapons during the first Gulf War [190], and there was an increased ALS incidence among returning veterans [191]. Thus, Sava et al. proposed that mycotoxin exposure could act as an environmental trigger associated with warfare that eventually causes humans to develop neurodegenerative diseases [192]. To demonstrate this, they administered mice with Ochratoxin A (OTA), another mycotoxin generated from Aspergillus that functions similarly to aflatoxins. Over the course of a two-week infusion period, animals were given cumulative doses of 4–16 mg/kg OTA by subcutaneously implanted minipumps. Both an increase in the brain’s anti-oxidative mechanisms and a dose-dependent decrease in striatal dopamine were noted. However, there were no signs of rigidity or delayed movements that would indicate Parkinsonism [193]. In contrast, following extensive behavioral examinations, PD-related behavior was discovered in another study looking into acute OTA poisoning. Therefore, it is possible to hypothesize that OTA exposure at low doses that coincide with the typical age-related drop in striatal dopamine (DA) may cause PD to manifest earlier than expected [194].

Alzheimer’s disease (AD): According to the infection theory associated with AD, individuals with AD have compromised immune systems and the BBB, making them more vulnerable to microbial infections that can lead to persistent neuroinflammation and neurodegeneration [195]. Further research into elevated levels of fungal polysaccharides and antifungal antibodies in AD patients’ blood sera, positive immunohistochemical staining, PCR tests, and fungal DNA sequencing from postmortem brains of AD patients provides more direct evidence [196]. Fungal species from *Botrytis*, *Candida*, *Alternaria*, *Fusarium*, *Cryptococcus*, *Cladosporium*, and *Saccharomyces* genera were discovered. Another investigation by the same group identified a subset of these fungal species in the CSF of AD patients [108,197]. Another study by the same group examined the CSF of AD patients and identified a subset of these fungal species [198]. The fungi’s mycotoxins may have exposed the host, although it remained unclear. As a result, more research will be needed to determine whether fungal infections and mycotoxins may have a causal involvement in AD. Further evidence suggests that some of these substances are even utilized to create mouse models of neurodegenerative disorders, which lends even more credence to the connection between mycotoxins and neurodegenerative diseases. For instance, the application of 3-nitropropionic acid (3-NP) is the basis of the HD model [199].

**Figure 6:**
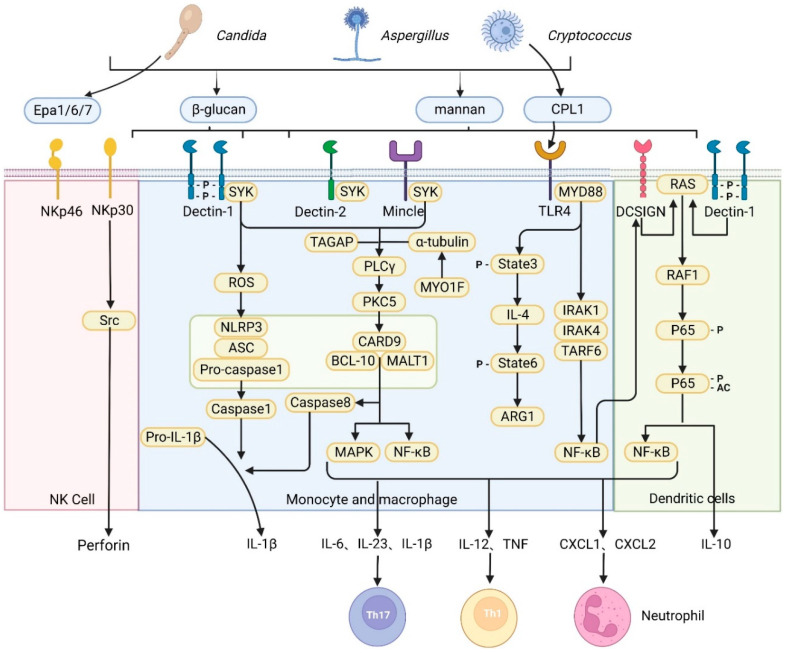
In fungal species, fungal carbohydrates are recognized by pattern recognition receptors (PRRs), triggering immunological responses. These receptors activate C-type lectin receptors (CLRs), initiate signaling pathways, and activate the Spleen Tyrosine Kinase (SYK)–CARD9 axis, which activates MAPK and NF-κB pathways, which produce chemokines and pro-inflammatory cytokines that support the development of CD4+ T cells. While dectin-1 activates non-canonical inflammasome activation through caspase-8, NLRP3 creates an inflammasome complex that generates IL-1β. Furthermore, Dendritic Cell-Specific Intercellular adhesion molecule-3-Grabbing Non-integrin (DC-SIGN) controls TLR signaling by causing dendritic cells’ NF-κB acetylation, while NK cells use their Natural Killer Cell p30-Related Protein and Natural Killer Cell p46-Related Protein (NKp30 and NKp46) receptors to identify the fungus. Reprinted from [203], Copyright © 2023 by the author and Frontiers In Immunology/Frontiers.

The role of PRRs and CARD9 signaling in fungus identification: Pattern recognition receptors (PRRs) that recognize pathogen-associated molecular patterns are the first step in initiating fungal infections, which are brought on by invading bacteria. Fungal infections are more likely due to mutations in the CARD9 gene, which have been connected to invasive fungal infections and persistent mucocutaneous candidiasis [200]. The pathogen Candida albicans depends on dendritic cells as its main source of PRR expression, which is essential for the pathogen to survive. PAMPs are present in many microorganisms, including Aspergillus and *Candida* species, and are located in the cell wall of *Candida albicans*. They are crucial for pathogen-host interactions [201]. Because *Aspergillus*, *Candida albicans*, and *Cryptococcus* differ in composition, the immune system might not recognize them. During germination, the chitin-rich wall of Aspergillus becomes hydrophobic, whereas the exopolysaccharide capsule of *Cryptococcus* stops PRRs from identifying its PAMPs. To effectively treat fungal infections, different PRRs set off the appropriate cellular responses and signals [202]. There are distinct PRRs that are expressed on various cells, and these PRRs can identify fungal invaders and activate efficient signaling pathways and cellular responses, according to Figure 6.

### 3.5. Direct Neurotoxicity

The term “neurotoxicity” describes the direct or indirect effects of substances that cause harm to an animal’s or a human’s nervous system. One of the strongest neurotoxins known to humans is botulinum toxin [204].

Botulinum-derived neurotoxins inhibit acetylcholine release at the peripheral neuromuscular junction (NMJ), leading to denervation and altered muscle tone. They strongly impede transmitter ablation and synaptic vesicle fusion [205]. BoNTs (Botulinum neurotoxins) also assist in keeping neurotransmitters other than ACh and other neurotransmitters in the NMJ. According to ample evidence, BoNTs also affect the PNS and CNS [206]. BoNTs in exocrine glands limit the release of CGRP, a cholinergic neurotransmitter that affects both cholinergic neuromuscular release and autonomic innervation, depending on the target tissue [207].

BoNTs are a viable treatment alternative since they provide benefits for disorders involving the release of excitatory neurotransmitters, which require muscle hyperactivity, and urological difficulties [208], or persistently uncomfortable circumstances [209], including migraines and headaches [210,211] and excruciating skeletal [212] or neuropathic states [213]. Despite multiple animal model studies showing their therapeutic potential, a major impediment prevents their clinical usage in people with cerebral neuronal hyperactivity-related CNS diseases. BoNTs are still the most powerful natural toxin, limiting their use in the brain for safety reasons. The BoNTs’ protein structure has two domains: one for binding and translocating, and the other for retaining the protease required to cleave the target Soluble NSF Attachment Protein Receptor (SNARE) proteins. This bi-chain structure allows the creation of chimeric proteins with binding and translocation domains engineered to reach well-defined CNS locations without the risk of botulinum neurotoxin (BoNT) systemic dissemination. Due to extensive research, several chimeric proteins have been created to re-target BoNTs to non-muscular sites [214,215,216,217,218,219,220,221,222,223,224].

**Figure 7:**
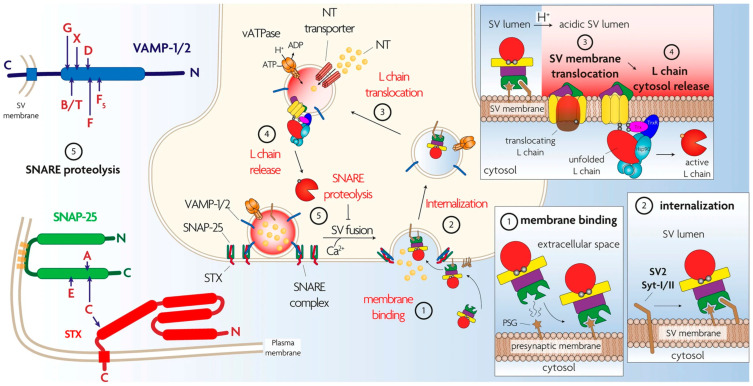
The complex relationship between neurotoxins and synaptic vesicle proteins highlights the critical function of the SNARE complex in neurotransmitter release and how the neurotoxins disrupt this biological process. 1. Binding: Using the C-terminal domain of the toxin, neurotoxins first bind to a polysialoganglioside molecule and a protein receptor on the cell surface. 2. Internalization: The neurotoxin is driven by this binding into the synaptic vesicle lumen, where it is acidified by a proton pump to promote the accumulation of neurotransmitters. 3. Translocation: With the aid of the chaperone Hsp90, the acidic environment causes a structural shift that allows the HN domain to pass through the membrane and translocate the metalloprotease domain into the cytoplasm. 4. Disulfide Bond Reduction: The NADH-Thioredoxin-Thioredoxin Reductase system inside the cytosol reduces the disulfide bond that binds the HN and metalloprotease domains, releasing the metalloprotease. 5. The metalloprotease cleaves a SNARE protein (VAMP, SNAP25, or Syntaxin) to stop neurotransmitter release and interfere with synaptic transmission. This process is known as SNARE Cleavage. Reprinted from [226] Copyright © 2022 by the author and Archives of Toxicology/Springer Nature.

The neuroparalytic disease known as botulism affects both humans and animals. It is caused by the botulinum neurotoxins that are generated by *Clostridium botulinum*, as well as by uncommon strains of *C. butyricum* and *C. baratii*. The capacity of botulinogenic clostridia to generate resistant endospores accounts for their widespread distribution in nature [225]. However, because BoNTs induce botulism, axonal transit from the PNS to the CNS may be lethal upon peripheral administration [205]. The most toxic proteins known to humans, botulinum neurotoxins, are a class of seven immunologically unique proteins (types A-G) generated by various strains of the anaerobic bacterium *Clostridium botulinum*. The molecular process comprises five steps that explain the neuroparalytic effects of BoNTs and tetanus neurotoxin (TeNT), as shown in Figure 7.

### 3.6. Mitochondrial Dysfunction and Apoptosis

Mitochondria, the power cells, may malfunction and cause many diseases. Mycotoxins, fungi’s secondary metabolites, may kill people and animals [227]. Citrinin, aflatoxin, and T-2 toxin cause mitochondrial dysfunction in test systems with multi-edged sword-like effects. Even at modest dosages, mycotoxins may produce oxidative stress, which may cause mitochondrial failure [228]. Low electron transport chain (ETC) efficiency and low ATP production characterize mitochondrial dysfunction [229]. The characteristics of aging are essential for all chronic diseases [230]. Mitochondrial disintegration is linked to neurodegenerative diseases such as Alzheimer’s, Parkinson’s, Huntington’s, and Friedreich’s ataxia [231]. Neurobehavioral and mental problems such as autism spectrum disorder, schizophrenia, bipolar disorder, and mood disorders are also dependent on mitochondrial dysfunction [232]. Mycotoxins are secondary metabolites, a structurally varied collection of generally low molecular weight chemicals produced by various molds or fungi [233]. In vitro, steroidogenesis in cultured adrenocortical carcinoma cells may need mitochondria and cholesterol sources. ACTH stimulates basal steroidogenic activity in cultured adrenocortical tumor cells, but moderate to high doses of cytochalasin B decrease both [234]. *Aspergillus ochraceus* produces ochratoxin A, a mycotoxin that is phytotoxic, teratogenic, nephrotoxic, and immunotoxic. Ochratoxin A (0.5 and 1 mM) and oosporein (0.25–1 mM) caused nephrotoxicity via lipid peroxidation or mitochondrial dysfunction. In isolated rat renal proximal tubules, ochratoxin A caused cell death and mitochondrial dysfunction [235]. However, it affected rat trachea Ca^2+^ homeostasis, presumably blocking mitochondrial enzymes and producing toxicity [236].

This mycotoxin also enhanced ROS, damaged mitochondria, and produced structural impairment in Arabidopsis thaliana [237]. Similar to how OTA causes an increase in respiration and a rise in the production of reactive oxygen species (ROS) inside the mitochondria, this also causes the opening of mitochondrial permeability transition pores that are reliant on ROS. As a result, cytochrome c was released into the cytosol, and the potential of the mitochondrial membrane decreased [238], causing an intrinsic apoptosis pathway.

**Figure 8:**
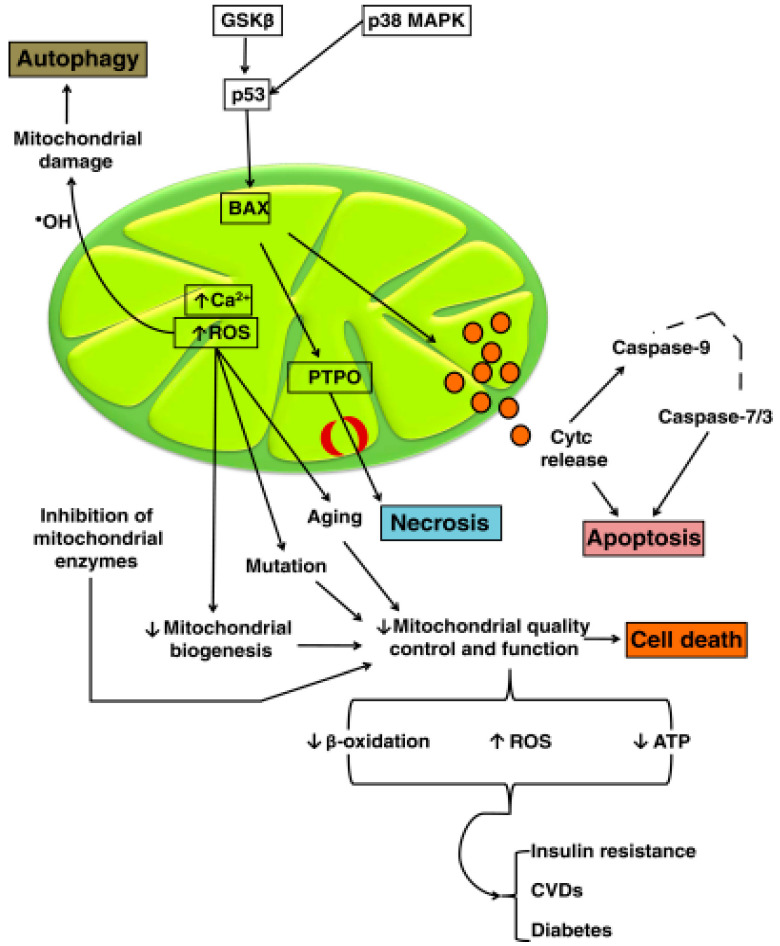
Molecular processes underlying mitochondrial malfunction and cellular events. Abbreviations: BAX (Bcl-2-associated X protein), ATP (adenosine triphosphate), CVDs (cardiovascular diseases), MAPK (mitogen-activated protein kinase), GSK3β (glycogen synthase kinase-3 beta), PTPO (permeability transition pore), and ROS (reactive oxygen species). Reprinted from [228]. Copyright @ 2018, International Union of Biochemistry and Molecular Biology.

Mammalian cells are susceptible to gliotoxin, a secondary metabolite produced by Aspergillus fumigatus. It causes the generation of ROS and the release of apoptogenic chemicals from the mitochondria, activating caspase-3 and inducing apoptosis via increasing the proapoptotic Bcl-2 family member Bak rather than Bax (Figure 8) [239]. Furthermore, this mycotoxin produced mitochondrial malfunction and ROS in Nicotiana tabacum BY2 cultured cells, resulting in transcriptional downregulation of the alternative oxidase (Aox1) gene and ion channel activity regulation, contributing to cell shrinkage. These pathways might have been implicated in activating programmed cell death [240].

The mycotoxin ochratoxin A (OTA), generated by Aspergillus and Penicillium subspecies, is a typical food and feed contaminant [175]. According to recent studies, OTA may also affect the neural system [241,242]. Based on rodent studies, OTA passes the BBB and accumulates in most brain areas over time and with concentration [192]. Both in vitro and in vivo evidence indicate the role of oxidative stress in OTA-mediated cytotoxicity [243,244,245,246,247,248].

The intricate processes by which OTA works encompass oxidative stress induction, mitochondrial dysfunction, bioenergetic compromise, protein synthesis suppression, DNA single-strand breakage, and OTA-DNA adduct formation [242,249]. The bioenergetic imbalance that is brought about by OTA may be the source of the development of free radicals and ROS, which in turn causes significant oxidative damage to DNA, lipids, and proteins via the synthesis of oxygen-free radicals and nitric oxide [250,251]. OTA causes oxidative stress, mitochondrial damage, and apoptosis, which are factors in neurological diseases like Parkinson’s and Alzheimer’s. Its effects include reduced DNA damage, decreased protein synthesis, and neurotoxicity, particularly in areas such as the hippocampus, which plays a critical role in neurodegeneration. Exposure to OTA has been associated with a connection to the pathogenesis of neurodegenerative diseases due to its potential to cause cognitive impairments, depression, and decreased neurogenesis [71].

### 3.7. Prion-like Behavior

**Figure 9:**
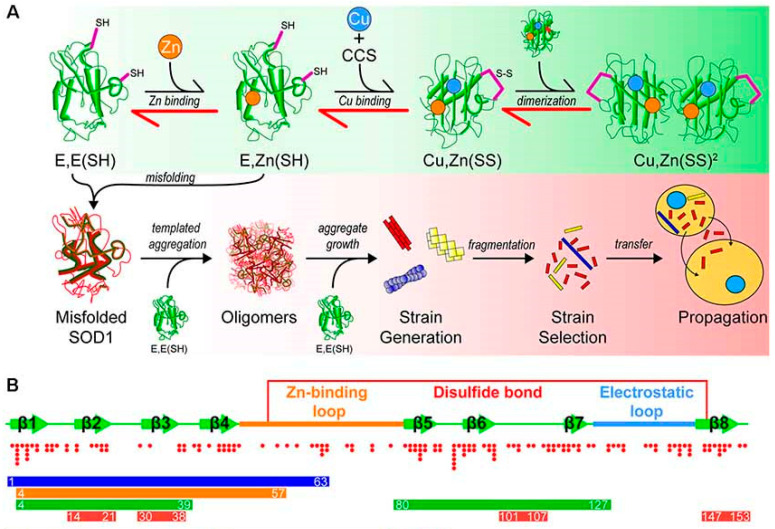
SOD1 native and off-folding pathways. (**A**) Native folding (green) yields a stable enzyme after metal binding; ALS-associated mutations (red) promote misfolding and aggregation. (**B**) SOD1 structure with mutation-prone regions highlighted.

According to Stanley B. Prusiner, the term “prion” refers to the infectious agent that causes transmissible spongiform encephalopathies (TSEs). Prusiner describes the prion as “a small proteinaceous infectious particle that is resistant to inactivation by most procedures that modify nucleic acids” [252]. Transmissible spongiform encephalopathies (TSEs) are a group of neurodegenerative diseases that are deadly in all cases. These disorders affect not just humans but also a wide variety of animals. TSEs are caused by an infectious agent known as the prion, which is a protein that is abnormally folded and aggregated. It spreads by imposing its form onto the host’s cellular prion protein (PrPC), which is responsible for the infection [73]. When scrapie prion protein (PrPSc) undergoes a structural shift from PrPC, it gives rise to a group of neurodegenerative diseases known as prion diseases. PrPC is involved in both the process of prion replication and prion-induced neurodegeneration [253]. There are undoubtedly multiple ways in which prions are different from all other known infectious diseases. First, it does not seem that prions have a genome of informative nucleic acids more extended than 50 bases that code for their offspring [254]. Second, a mutated version of the cellular prion protein, PrPC, encoded by the gene Prnp, is the only known component of the prion. PrPC is a cell surface glycoprotein [255] of unknown function that has been found in all Xenopus laevis species as well as all mammals and birds [256] and fish [257]. Third, the structural transformation of PrPC into PrPSc, an insoluble, largely protease-resistant isoform that spreads by forcing its aberrant conformation onto PrPC molecules, is the key event in prion disease. Several studies have demonstrated the significance of PrPC in both prion replication and prion-induced neurodegeneration [258]. According to the findings, transgenic animals expressing solely a secreted version of PrPC missing a glycosylphosphatidylinositol (GPI) anchor do not develop clinical indications of prion disease, despite the fact that prion inoculation stimulates PrPSc production and amyloid plaque aggregation [259]. Prion disease is characterized by extensive neurodegeneration; therefore, affected individuals show clinical symptoms of both cognitive and motor impairment. In addition, the disease is defined by the spread of infectious prions and, in many cases, the production of amyloid plaques [73]. Prion-like amyloids, like yeast, can also be seen in lower eukaryotes [260]. Eukaryotic Polypeptide Chain Release Factor 3 (eRF3), also known as Sup35p (eukaryotic translation release factor of *S. cerevisiae*), is the translation termination factor in yeast. The most well-studied yeast prions are the [PSI+] prion yeast from Saccharomyces cerevisiae and the [PSI+] prion state of eRF3 [261]. The percentage at which PrPSc forms and, thus, the duration of the incubation period appear to be inversely correlated with the degree of PrPC expression. Prnpo/o mice do not spread the scrapie virus and are resistant to prions [262]. When Sup35p is transformed into the [PSI+] prion, its function is diminished, leading to an incorrect translation termination process. As a consequence, the prion form causes proofreading of mRNA termination codons. Polypeptides with longer C-termini and altered functions were assumed to be produced by [PSI+], increasing the phenotypic plasticity of yeast and its resistance to harsh conditions [263]. Various prion-forming domains can be encoded by the yeast genome, resulting in various prion protein variations or phenotypes. As seen in Figure 9, most yeast prions are self-replicating [264,265].

Owing to particular theories, prion-like mechanisms have a role in neurodegenerative diseases like ALS, Parkinson’s, and Alzheimer’s. These disorders are thought to be caused by aberrant proteins like tau, αSyn, and TDP-43, which change normal proteins into harmful variants and spread cell-to-cell in the brain [266]. This prion-like propagation might explain the degeneration of subsets of neurons, the various but characteristic pathologies, and the disease’s development. It is the fundamental molecular mechanism of the many major neurodegenerative disorders with amyloid-like aberrant protein pathologies [267]. Thus, controlling the spread of aberrant proteins is a crucial objective for treating these severe neurodegenerative diseases.

This concept describes how superoxide dismutase 1 (SOD1) aggregates and propagates like a prion in ALS in Figure 9. SOD1 grows in the native folding process (green) by binding Zn and Cu, resulting in a stable enzyme via dimerization. In the off-folding route (red), ALS-related mutations cause misfolding, driving SOD1 into unstable phases. These misfolded proteins recruit more SOD1, resulting in oligomers that assemble. These aggregates may acquire strain-like qualities, and fragments of these strains can spread to neighboring cells, causing additional misfolding.

## 4. Conclusions and Future Perspectives

In conclusion, scientists are becoming increasingly aware of the intricate relationship between neurodegenerative diseases and microbial toxins. The slow loss of neurons and synapses in neurodegenerative disorders threatens global health. Microbial toxins, produced by many bacteria and fungi, have offered new insights into these diseases. In addition to infecting, these poisons may cause neurodegeneration. Academic research suggests that microbial toxins may affect brain homeostasis by activating inflammatory pathways, oxidative stress, and protein misfolding. Gram-negative bacteria generate LPS that may cause CNS inflammation and neuron damage. Neurodegenerative protein aggregation has also been related to fungal toxins like Candida albicans. These proteins include Alzheimer’s amyloid-beta and PD α-Syn. Multiple neurodegenerative diseases have been linked to gut microbiota dysbiosis. General health depends on the gut microbiome. Altering gut microbiota may boost the generation of toxins and chemicals that compromise the gut barrier, increasing intestinal permeability. This condition makes pro-inflammatory molecules easier to circulate, which may affect the brain and worsen neurodegeneration. In particular, neurodegenerative diseases commonly cause gastrointestinal symptoms, demonstrating the link between gut health and nervous system function. Another factor is that microbial toxins may contribute to neurodegenerative processes via various mechanisms. For instance, bacterial toxins activate immune system PRRs, triggering an inflammatory cascade that damages brain structures. This approach emphasizes the immune regulation’s potential to reduce microbial pathogen neurotoxicity. Innovative therapies that boost immune response or misfolded protein clearance may be promising.

Nanotechnology and gene therapy provide new opportunities for personalized therapies. These methods directly transport therapeutic medications to wounded central nervous system locations, bypassing the blood–brain barrier. Immunotherapy may also control microbial toxic immunological responses, lowering neuroinflammation and maintaining neuronal function. Infections produced by bacteria and fungi combined may be serious. Some CNS fungal species cohabit with bacteria. These communities’ interactions may boost neurotoxic drug effects. Understanding the synergistic relationships between bacteria may illuminate novel disease development pathways and provide intervention options. This debate must also include the significance of mitochondrial dysfunction in neurodegenerative diseases. Many mycotoxins and bacterial toxins affect mitochondrial function, causing oxidative stress and neuron loss. Addressing mitochondrial health via lifestyle modifications, dietary therapies, and pharmacological methods may help control neurodegenerative diseases.

Neurology, immunology, and microbiology must be combined to understand these complex interactions. Further research will reveal how microbial poisons affect neurodegenerative processes, which may inspire new preventative and therapeutic methods. Environmental, microbiological, and biological factors must be addressed to improve patient outcomes and quality of life for neurodegenerative disease patients. If we understand these links, we may develop novel neurodegenerative treatments and stay at the forefront of research and therapy. Toxin-induced neurodegeneration’s molecular pathways and particular microbial toxins that cause disease pathology need additional study. High-throughput sequencing, metabolomics, and computational modeling will enable gut microbiota composition and toxin production dynamics characterization in health and illness. These technologies will allow researchers to detect disease-risk microbial biomarkers, assess therapy responses, and create patient-specific therapeutic regimens. Microbiologists, neuroscientists, immunologists, and doctors must collaborate to advance this discipline. Preclinical animal models and humanized microbiota systems will illuminate the links between microbial toxins and neurodegenerative disorders. Probiotic, nutritional, and FMT clinical studies must provide evidence-based treatment recommendations.

## Figures and Tables

**Table 2:** Examples of mycotoxins triggering different physiological responses.

Mycotoxin	Cell Culture Model	Assay	Mechanism of Action	Level of Evidence		References
Patulin	Mouse enteric neurons SH-SY5Y	Measurement of neurite outgrowth. Calcium flux analysis using Calbryte 520 glucose-Glo assay The Cell Titer-Glo Assay The ROS-Glo H_2_O_2_ assay	Reduced viability and ROS generation in SH-SY5Y. Reduced overall neurite mass, elevated calcium entry, decreased viability, and decreased cellular glucose concentration in enteric neurons	Low to Moderate evidence		[169]
Fumonisin B_1_	Mouse astrocytes BV-2 N2a Mouse cortical neurons	3-(4,5-dimethylthiazol-2-yl)-2,5-diphenyltetrazolium bromide (MTT) assay Propidium iodide + Annexin V staining qPCR Thymidine incorporation Lactate dehydrogenase assay	Reduced viability and necrotic cell death in BV-2-infected astrocytes. Minimized BV-2 proliferation. Downregulated TNFα and IL-1β in BV-2+ astrocytes. No alterations in cortical neurons or N2. Reduced BV-2 proliferation.	Low to Moderate evidence		[170]
Lolitrem B	HEK293	Electrophysiology	Suppressed potassium currents in the hSlo channel across various depolarizing voltages in a concentration-dependent way.	Low evidence		[171]
Patulin	HEK293	Transcriptome and proteome profiles using digital gene expression (DGE) and isobaric tagging (iTRAQ). Cell Counting Kit-8 Lactate dehydrogenase assay. The MTT assay	Alterations in the expression of genes or proteins linked to the cell cycle, oxidative phosphorylation, ribosome, and apoptosis Cell death is mediated by caspase through an inherent apoptotic pathway Cytochrome C is released into the cytosol from mitochondria.	Moderate evidence		[172]
Ochratoxin A	HT22 SH-SY5Y	The 2′,7′-Dichlorofluorescein (DCF) test generates reactive oxygen species (ROS). Proteomic study using 2-DE gels. Lactate dehydrogenase assay. Hoechst staining and Western blot analysis. The MTT assay	Overexpression of proteins linked to the etiology of neurodegenerative diseases in HT22 SH-SY5Y + HT22 showed decreased viability and elevated oxidative stress. Increased p53 phosphorylation and caspase activation in HT22		Moderate evidence	[173]
Penitrem A	Rat granule neurons in the cerebellum	ROS generation by DCF assay MTT assay	Increased ROS production. Cell survival decreases with time and concentration	Moderate evidence		[174]
Ochratoxin A	Rat cortical neurons SH-SY5Y	Mitochondrial membrane potential assessment by JC-1 staining DNA fragmentation assay Western blot analysis Neutral red assay	Reduced number of cells, fewer neurites, and some tubercles in primary neurons. Reduced potential of the mitochondrial membrane. Reduced viability in cortical neurons expressing SH-SY5Y and increased sensitivity in primary neurons. Apoptosis triggered by caspases in cortical neurons and SH-SY5Y	Moderate evidence		[175]
Penitrem A	Rat cerebellar synaptosomes	Assay for [3H] GABA + [3H] glutamate absorption and lactate dehydrogenase activity.	The reduction of neurotransmitter uptake was not caused by hole formation or disruption of the plasma membrane, as evidenced by the unchanged levels of the cytosolic marker lactate dehydrogenase. inhibition of [3H] GABA + [3H] glutamate’s high affinity uptake		Moderate evidence	[176]

## Data Availability

Data are contained within the article.

## Abbreviations

LPS	Lipopolysaccharide
BBB	blood–brain barrier
AD	Alzheimer’s disease
PD	Parkinson’s disease
HD	Huntington’s disease
ALS	amyotrophic lateral sclerosis
PRRs	express pattern recognition receptors
TLRs	toll-like receptors
ROS	reactive oxygen species
GI	gastrointestinal
α-Syn	Alpha-synuclein
ASD	Autism spectrum disorder
IL-10	Interleukin-10
IL-6	interleukin-6
IL-17	interleukin-17
IFN-γ	interferon-gamma
MS	Multiple sclerosis
LTA	lipoteichoic acid
TLR	toll-like receptor
6-OHDA	6-hydroxy-dopamine
MDMA	4-methylenedioxymethamphetamine
DA	dopamine
CNS	central nervous system
O_2_	Superoxide
H_2_O_2_	hydrogen peroxide
NO	nitric oxide
PN	peroxynitrite
NADPH	Nicotinamide Adenine Dinucleotide Phosphate Hydrogen
APP	amyloid precursor protein
TLR2	Toll-like receptor 2
TLR1	Toll-like receptor 1
CD14	cluster of differentiation 14
MG	methylglyoxal
FB1	fumonisin B1
CSF	cerebrospinal fluid
PCR	polymerase chain reaction
TDP-43	transactive response DNA binding protein 43
Cu/Zn-SOD1	Copper/Zinc Superoxide Dismutase 1
OTA	Ochratoxin A
3-NP	3-Nitropropionic
PRRs	Pattern recognition receptors
PAMPs	pathogen-associated molecular patterns
CARD9	Caspase Recruitment Domain-containing protein 9
CLRs	C-type lectin receptors
SYK	Spleen Tyrosine Kinase
DC-SIGN	Dendritic Cell-Specific Intercellular adhesion molecule-3-Grabbing Non-integrin
NKp30	Natural Killer Cell p30-Related Protein
NKp46	Natural Killer Cell p46-Related Protein
BoNTs	botulinum neurotoxins
NMJ	neuromuscular junction
Ach	acetylcholine
PNS	peripheral nervous systems
CGRP	Calcitonin Gene-Related Peptide
SNARE	Soluble NSF Attachment Protein Receptor
TeNT	tetanus neurotoxins
ETC	electron transport chain
ATP	adenosine-5′-triphosphate
ACTH	Adrenocorticotropic Hormone
Aox1	alternative oxidase
BAX	Bcl-2-associated X protein
CVDs	cardiovascular diseases
GSK3β	glycogen synthase kinase-3 beta
MAPK	mitogen-activated protein kinase
PTPO	permeability transition pore
TSEs	transmissible spongiform encephalopathies
PrP^C^	cellular prion protein
PrPSc	scrapie prion protein
GPI	glycosylphosphatidylinositol
eRF3	Eukaryotic Polypeptide Chain Release Factor 3
SOD1	superoxide dismutase 1
